# Real-time and nested polymerase chain reaction in the diagnosis of multifocal serpiginoid choroiditis caused by *Mycobacterium tuberculosis* - a case report

**DOI:** 10.1186/s12348-014-0029-5

**Published:** 2014-11-18

**Authors:** Sachin B Shetty, Jyotirmay Biswas, Sowmiya Murali

**Affiliations:** Vitreo-Retina & Uvea Services, Sadguru Netra Chikitsalaya, Jankikund Post, Chitrakoot, 210204 U.P. India; Uveitis & Ocular Pathology Department, Vision Research Foundation, 18, College Road, Nungambakkam, Chennai, 600006 Tamil Nadu India; Medical Research Foundation, 18, College Road, Sankara Nethralaya, Nungambakkam, Chennai, 600006 Tamil Nadu India

**Keywords:** Multifocal serpiginoid choroiditis, Serpiginous choroiditis, Real-time PCR, Nested PCR

## Abstract

**Background:**

The term multifocal serpiginoid choroiditis (MSC) has been proposed for the infective variant of serpiginous choroiditis (SC) to distinguish it from typical SC believed to be autoimmune related. The role of *Mycobacterium tuberculosis* (MTb) in MSC has been studied by conventional polymerase chain reaction (PCR). However, the use of real-time PCR (RT-PCR) and nested PCR (N-PCR) in MSC has not been reported. This paper aims to highlight the usefulness of PCR in identifying MTb as a causative agent for MSC leading to its correct treatment with anti-tubercular therapy (ATT).

**Findings:**

A young male with a family history of tuberculosis (TB) presented with a history of diminution of vision (DOV) since 3 months in his right eye (RE). He gave similar history in his left eye (LE) since 3 years. His fundus findings were suggestive of MSC. His high-resolution computed tomography (HRCT) chest and Quanti-FERON TB gold results were positive for MTb. These suggested TB to be the likely cause for MSC. This was confirmed by a positive N-PCR report of his aqueous specimen. Further RT-PCR was done to quantify the bacillary load before starting therapy. He was advised 9 months of ATT with 6 weeks of oral steroids. At last follow-up, the RE showed better healing than the LE with fewer chorioretinal scars and a better visual acuity.

**Conclusions:**

RT and N-PCR for MTb are useful in establishing a tuberculous etiology in MSC. Coupled with a good response to ATT, these tests justify the use of ATT in MSC with a PCR-confirmed MTb report.

**Electronic supplementary material:**

The online version of this article (doi:10.1186/s12348-014-0029-5) contains supplementary material, which is available to authorized users.

## Findings

### Introduction

Serpiginous choroiditis (SC) is used to describe a bilateral chronic recurrent geographic pattern of choroiditis that typically extends from the peripapillary area and affects the overlying retinal pigment epithelium (RPE) and outer retina [1]. Autoimmune [2], infective [3], vascular, and degenerative mechanisms have all been proposed in its etiopathogenesis. The infectious type of SC is mostly commonly associated with tuberculosis (TB). Other causes include herpes viruses, syphilis, fungi, and toxoplasma [4]. Recently, Nazari and Rao [4] proposed the term multifocal serpiginoid choroiditis (MSC) for the infective variant of SC to distinguish it from typical SC.

### Background

A study of MSC patients with polymerase chain reaction (PCR) by Mohan et al. [5] concluded that *Mycobacterium tuberculosis* (MTb) (53.8 %) was the most common and important etiologic agent for MSC in TB endemic areas. One of the drawbacks of their study was that DNA copy numbers were not assessed by real-time PCR (RT-PCR). Annamalai et al. [6] used RT-PCR in 29 cases of typical SC and demonstrated MTb DNA in only 1 case, thus concluding a chance/rare association of TB with SC. In this series, nested PCR (N-PCR) was not performed in the positive case.

Herein, we report a case of MSC which was proved to be caused by MTb by both RT-PCR and N-PCR. To our knowledge, this is the first case report where both RT-PCR and nested PCR were used to show an association between TB and MSC.

### Case description

A 31-year-old male from Central India presented with a history of painless progressive diminution of vision (DOV) with floaters since 3 months in his right eye (RE). He was treated elsewhere with a short course of oral steroid over 1 month. Since 6 days, he noticed a further drop in his RE vision and was referred to us. He also had a similar history of sudden DOV in his left eye (LE) which was untreated and has been constant since 3 years. He had a family history of TB with both of his parents treated for the same.

**Figure 1:**
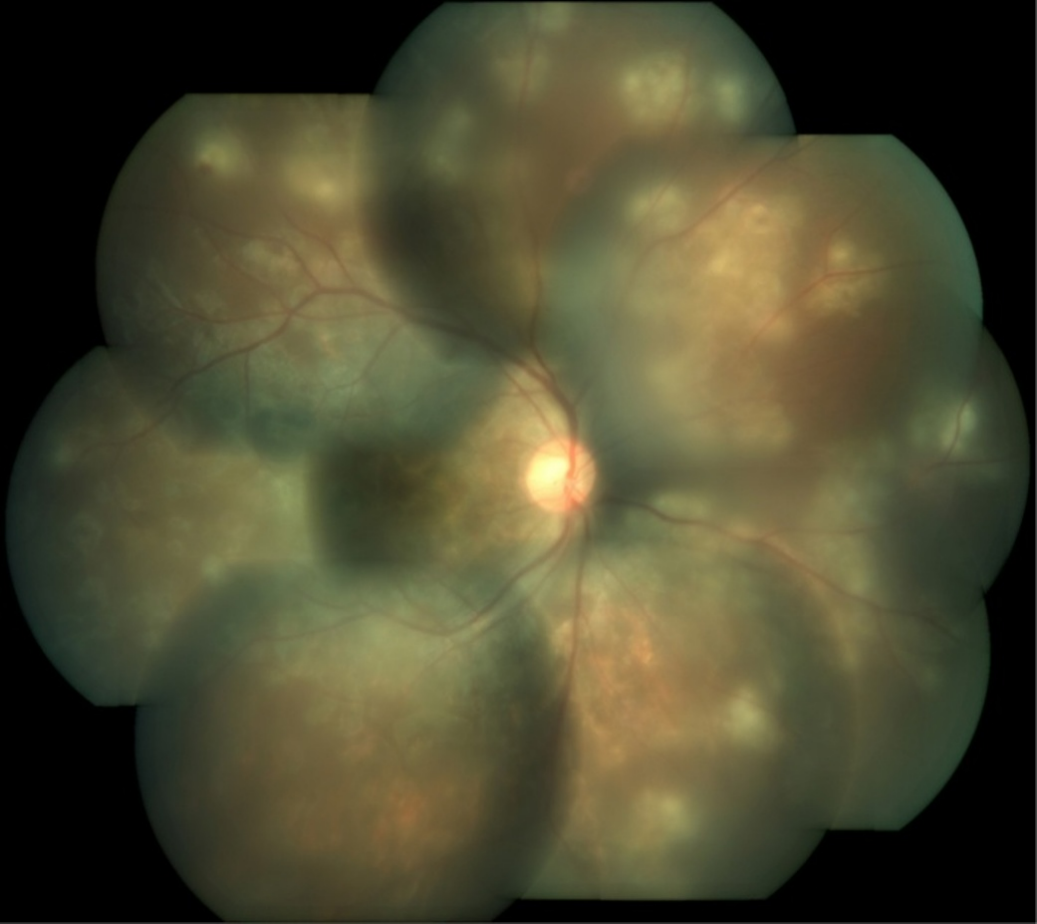
**Active multifocal serpiginoid choroiditis.** Montage picture of the right eye showing multiple discrete areas of active choroiditis amidst areas of healed chorioretinal atrophic scars with grade 2 vitritis at presentation.

**Figure 2:**
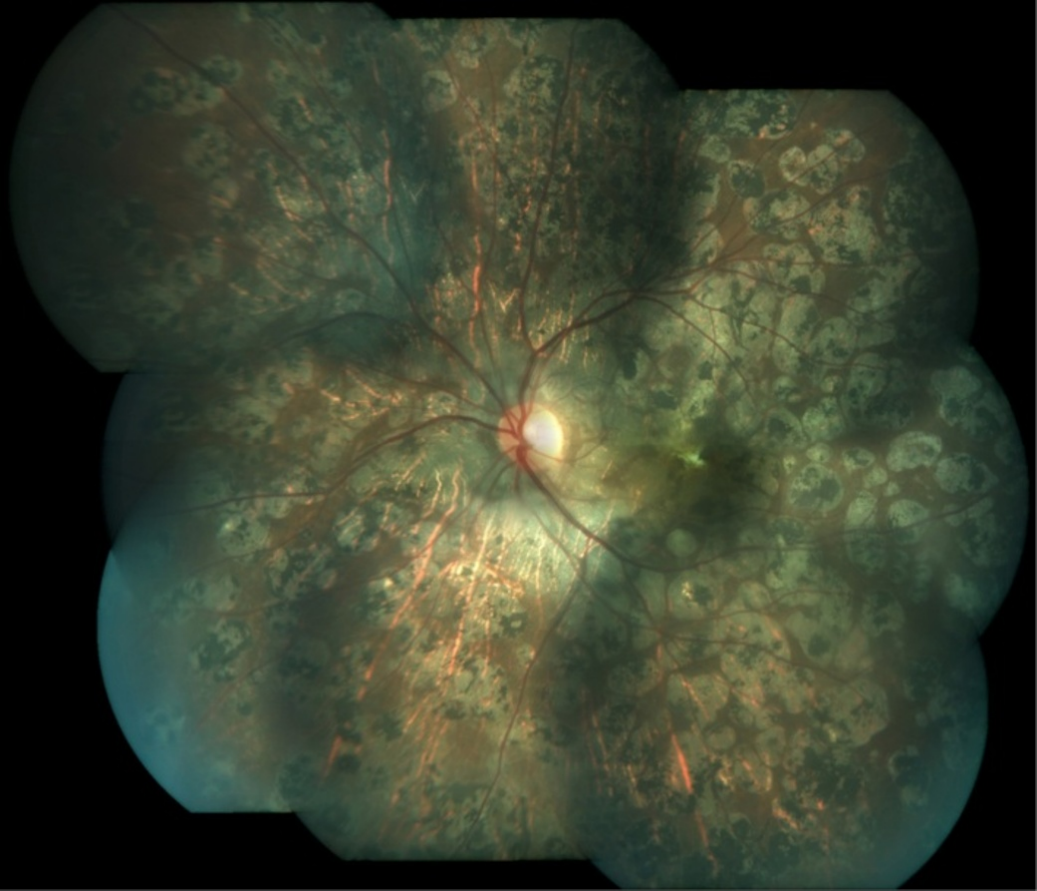
**Healed multifocal serpiginoid choroiditis.** Montage picture of the left eye showing multiple healed chorioretinal scars with subretinal fibrosis at the macula at presentation.

**Figure 3:**
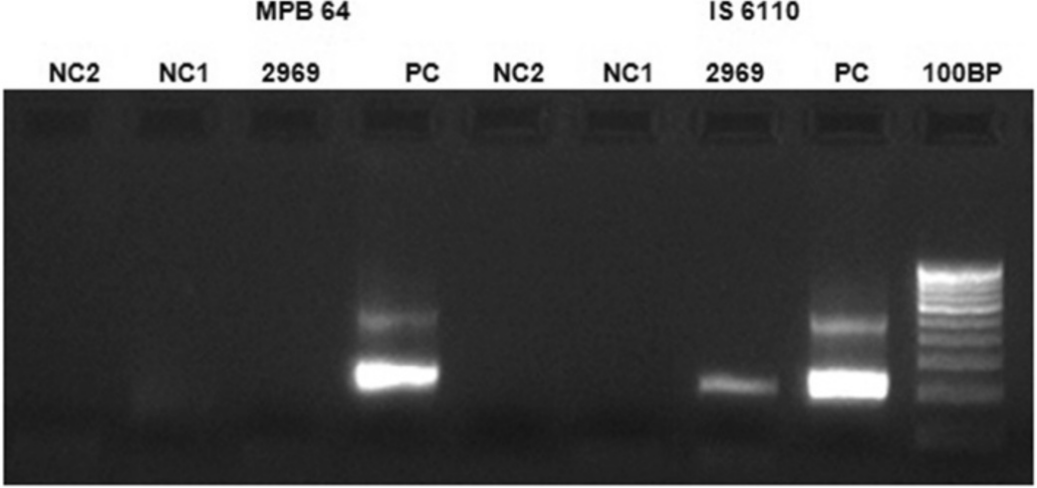
**Image showing agarose gel picture of the PCR amplification.** Lane 1: negative control 2 for MPB 64. Lane 2: negative control 1 for MPB 64. Lane 3: aqueous aspirate of the patient negative for MPB 64. Lane 4: positive control for MPB 64. Lane 5: negative control 2 for IS 6110. Lane 6: negative control 1 for IS 6110. Lane 7: aqueous aspirate of the patient positive for IS 6110. Lane 8: positive control for IS 6110. Lane 9: molecular weight - 100-bp ladder.

**Figure 4:**
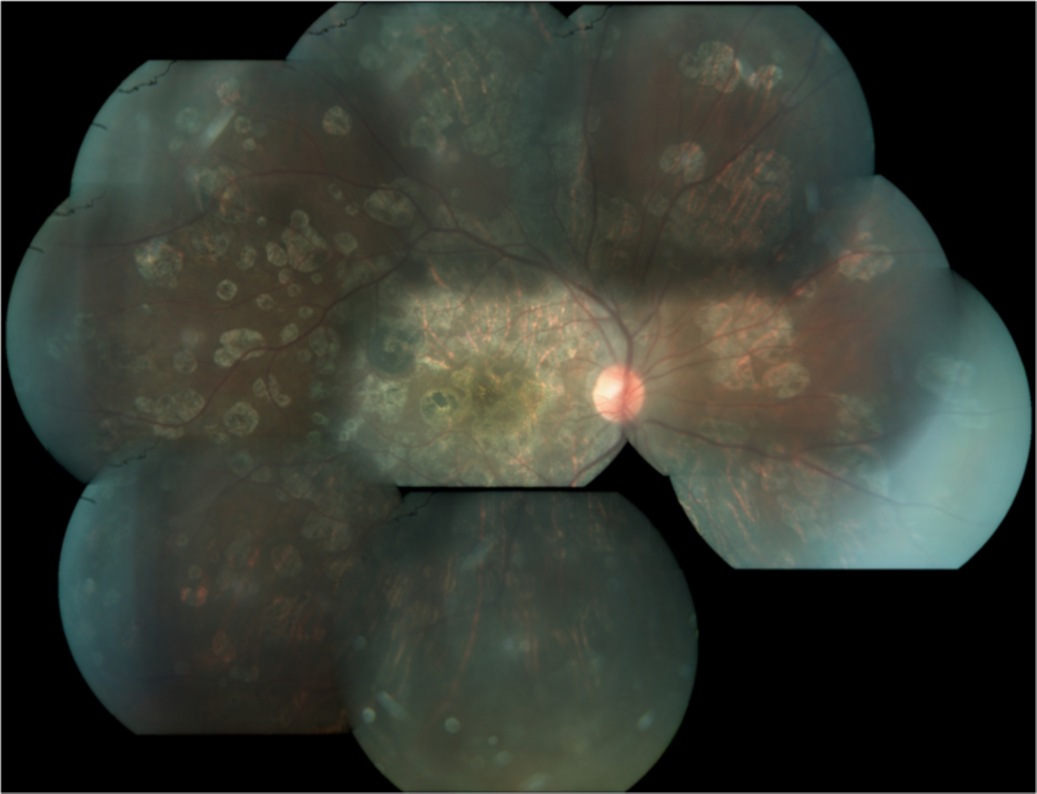
**Healed multifocal serpiginoid choroiditis.** Montage picture of the right eye showing resolution of active choroiditis leaving behind multiple healed chorioretinal scars at the end of the fourth month after initiation of antitubercular therapy.

**Figure 5:**
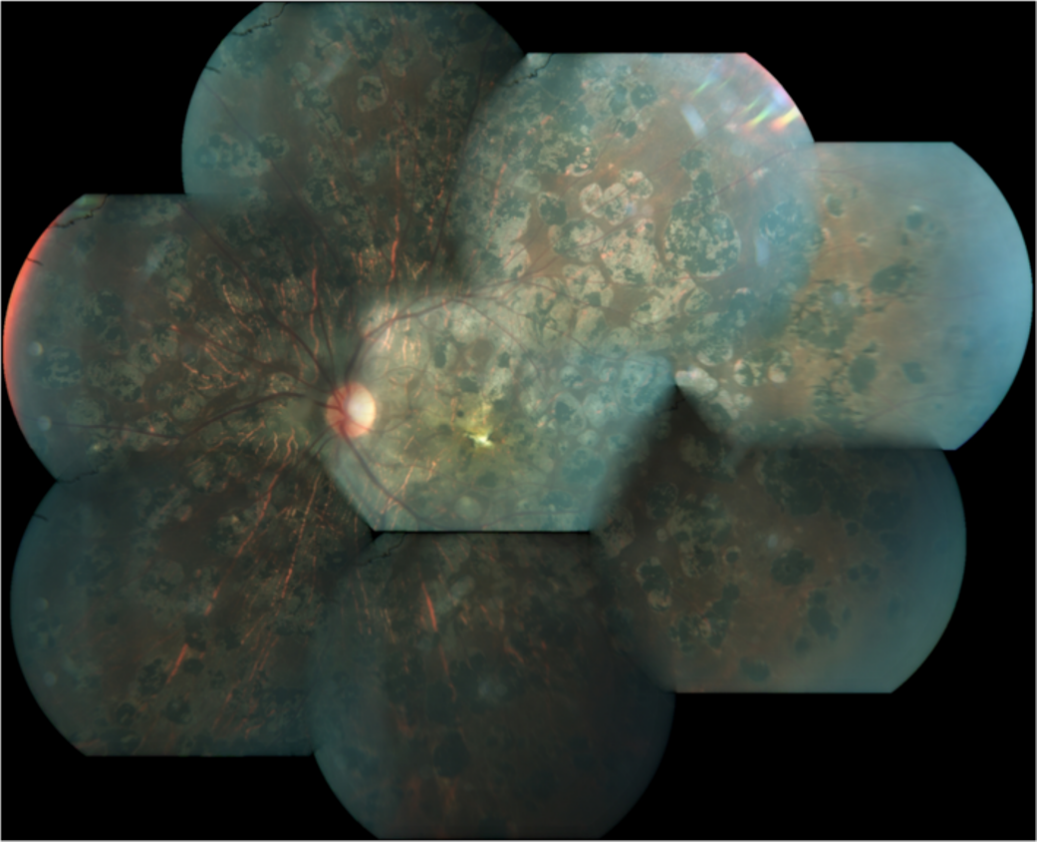
**Healed multifocal serpiginoid choroiditis.** Montage picture of the left eye showing multiple healed chorioretinal scars with subretinal fibrosis at the macula at the fourth month follow-up.

On examination, the best corrected visual acuity (BCVA) in the RE was 3/60 (20/400), <N36, and in the LE was counting fingers close to face (CFCF), <N36 for distance and near, respectively. Anterior segment and intraocular pressures were normal in both eyes (BE). Fundus evaluation of the RE showed multiple discrete areas of active choroiditis amidst areas of chorioretinal atrophic (CRA) scars and vitritis grade 2 (Figure 1). The LE showed occasional vitreous cells and multiple healed CRA scars all over the fundus with subretinal fibrosis involving the macula (Figure 2). High-resolution computed tomography (HRCT) chest showed tiny multiple mediastinal adenopathy suggestive of TB. Quanti-FERON TB gold test (Cellestis Limited, Carnegie, Victoria, Australia) was positive. Rapid plasma reagin and *Treponema pallidum* hemagglutination assay were negative. These features were suggestive of MSC secondary to TB in BE with reactivation in the RE. The etiological diagnosis was confirmed by positive N-PCR (Figure 3) and RT-PCR report of aqueous specimen (Additional file 1). After a physician clearance, the patient was advised a 9-month course of antitubercular therapy (ATT) with a tapering course of oral steroids over 6 weeks. At his last follow-up, at 4 months after initiating ATT, his right eye had a BCVA of 20/125 (6/36) with healed chorioretinal scars (Figure 4 and Figure 5).

### Discussion

This case report demonstrates both RT and N-PCR positivity in a case of MSC, establishing tuberculous etiology conclusively. It justifies the use of ATT in MSC patients with a PCR-confirmed MTb report. Under ATT cover, the patient's RE healed better than the LE with fewer chorioretinal scars and a better visual acuity (20/125 versus CFCF). Not only is ATT the definitive and specific treatment in these cases, but it also prevents recurrence of uveitic attacks [7].

RT-PCR can detect, amplify, and quantify the bacillary load in real time thus giving an idea about the disease severity. Serial monitoring of RT-PCR after starting treatment may show reduced number of DNA copies, thus indicating a beneficial response to therapy. In this case, however, due to ethical, logistical, and lack of patient's consent, we could not repeat RT-PCR during the follow-up visits. Hence, we could not correlate RT-PCR with disease severity or treatment response to ATT. Properly designed prospective studies may demonstrate this correlation.

Nested PCR [8] is a modification of conventional PCR intended to reduce contaminations in products due to amplification of unexpected primer binding sites. RT-PCR is much more expensive and complicated as compared to N-PCR. Hence, N-PCR could be useful initially in establishing the etiological diagnosis and then following it up with RT-PCR later to assess the treatment response. Further studies may help in incorporating PCR (wherever feasible) in the local practice guidelines before prescribing ATT for the MSC cases. This may limit the unwarranted use of ATT and its side effects in non-tuberculous choroiditis.

However, a negative PCR from anterior chamber (AC) tap theoretically cannot rule out tuberculous etiology in MSC as AC tap may often be negative as compared to vitreous tap in posterior segment inflammation. A large prospective study is needed to determine the sensitivity and specificity of AC tap.

## Consent

Written informed consent was obtained from the patient for the publication of this report and any accompanying images.

## Additional file

## Electronic supplementary material

Additional file 1:**PCR: quantitation report.** Quantitation data for Cycling A.Green and standard curve. (DOCX 69 KB)

Below are the links to the authors’ original submitted files for images.

Authors’ original file for figure 1

Authors’ original file for figure 2

Authors’ original file for figure 3

Authors’ original file for figure 4

Authors’ original file for figure 5

## Abbreviations

ATT: antitubercular therapy
CRA: chorioretinal atrophic
DOV: diminution of vision
HRCT: high-resolution computed tomography
LE: left eye
MSC: multifocal serpiginoid choroiditis
MTb: *Mycobacterium tuberculosis*
N-PCR: nested PCR
PCR: polymerase chain reaction
RE: right eye
RT-PCR: real-time PCR
SC: serpiginous choroiditis
TB: tuberculosis
