# Supplementary material for: Real-time and nested polymerase chain reaction in the diagnosis of multifocal serpiginoid choroiditis caused by Mycobacterium tuberculosis - a case report
Source: J Ophthalmic Inflamm Infect. 2014 Nov 18;4:29. doi: 10.1186/s12348-014-0029-5 (PMC4884041; doi:10.1186/s12348-014-0029-5)
Supplement: Supplementary file 1 — Additional file 1:PCR: quantitation report. Quantitation data for Cycling A.Green and standard curve. (DOCX 69 KB) [file 12348_2014_29_MOESM1_ESM.docx]

| 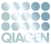 |  |
| --- | --- |

# PCR : Quantitation Report

#### Quantitation data for Cycling A.Green


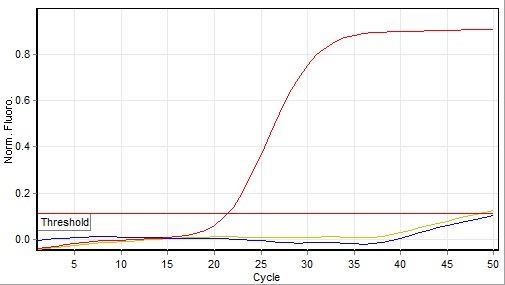


#### Standard Curve


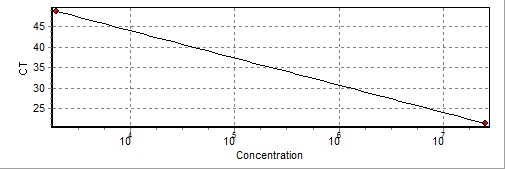


| No. | Colour | Name | Type | Ct | Given Conc (copies/ml) | Calc Conc (copies/ml) | % Var |
| --- | --- | --- | --- | --- | --- | --- | --- |
| 1 | 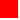 | S1 | Standard | 21.44 | 25,000,000 | 25,000,000 |  |
| 2 | 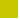 | VRF NO 2969/14 | Unknown | 48.65 |  | 1,966 |  |
| 3 | 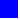 | NC | NTC |  |  |  |  |

**Legend:**
NEG (NTC) - Sample cancelled due to NTC Threshold.
NEG (R. Eff) - Sample cancelled as efficiency less than reaction efficiency threshold.

| This report generated by Rotor-Gene Q Series Software 2.0.2 (Build 4) Copyright 2008 Corbett Life Science, a QIAGEN Company. All rights reserved. ISO 9001:2000 (Reg. No. QEC21313) 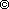 |
| --- |
