# Supplementary figures and images for: Real-time and nested polymerase chain reaction in the diagnosis of multifocal serpiginoid choroiditis caused by Mycobacterium tuberculosis - a case report
Source: J Ophthalmic Inflamm Infect. 2014 Nov 18;4:29. doi: 10.1186/s12348-014-0029-5 (PMC4884041; doi:10.1186/s12348-014-0029-5)

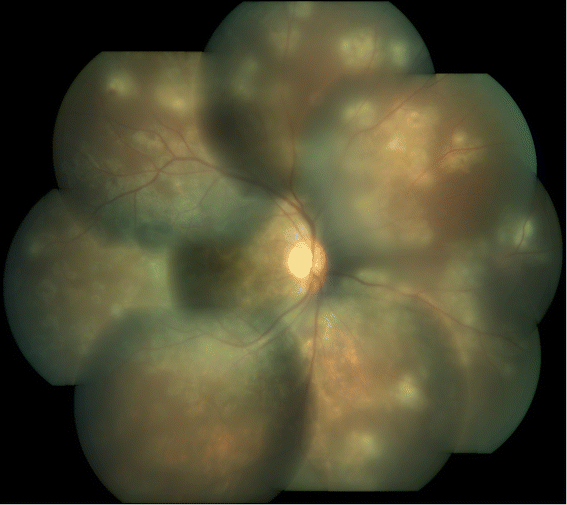

Supplement: Supplementary file 2 — Authors’ original file for figure 1 [file 12348_2014_29_MOESM2_ESM.gif]

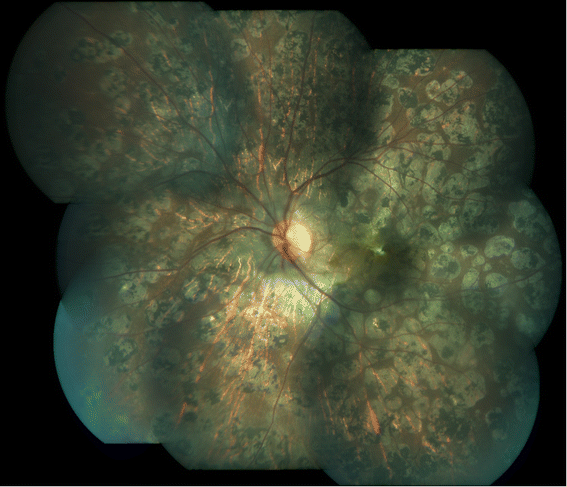

Supplement: Supplementary file 3 — Authors’ original file for figure 2 [file 12348_2014_29_MOESM3_ESM.gif]

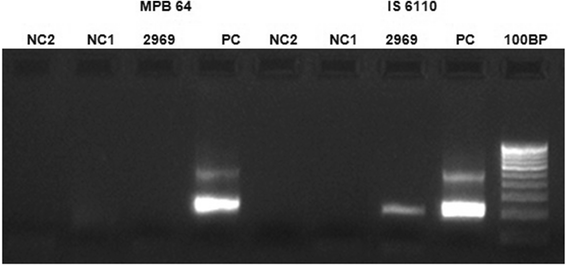

Supplement: Supplementary file 4 — Authors’ original file for figure 3 [file 12348_2014_29_MOESM4_ESM.gif]

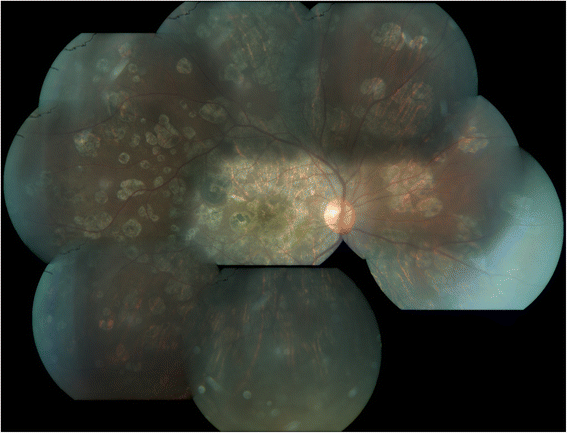

Supplement: Supplementary file 5 — Authors’ original file for figure 4 [file 12348_2014_29_MOESM5_ESM.gif]

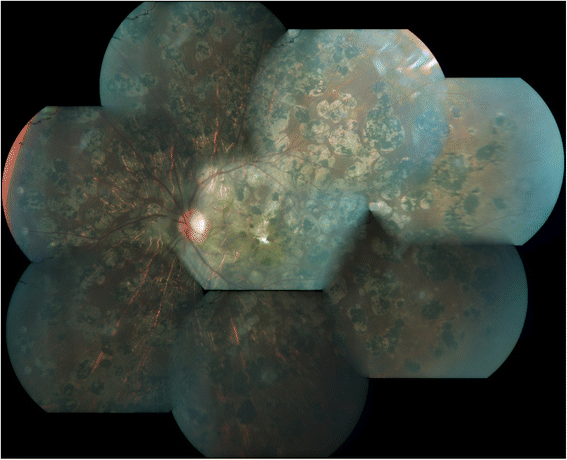

Supplement: Supplementary file 6 — Authors’ original file for figure 5 [file 12348_2014_29_MOESM6_ESM.gif]
